# Supplementary material for: Surface cooling caused by rare but intense near-inertial wave induced mixing in the tropical Atlantic
Source: Nat Commun. 2020 Jul 31;11:3829. doi: 10.1038/s41467-020-17601-x (PMC7395095; doi:10.1038/s41467-020-17601-x)
Supplement: Supplementary file 1 — Supplementary Information [file 41467_2020_17601_MOESM1_ESM.pdf]

Supplementary Material

Surface cooling caused by rare but intense near-inertial wave induced mixing in the tropical Atlantic

Hummels et al.

Supplementary Figure 1:

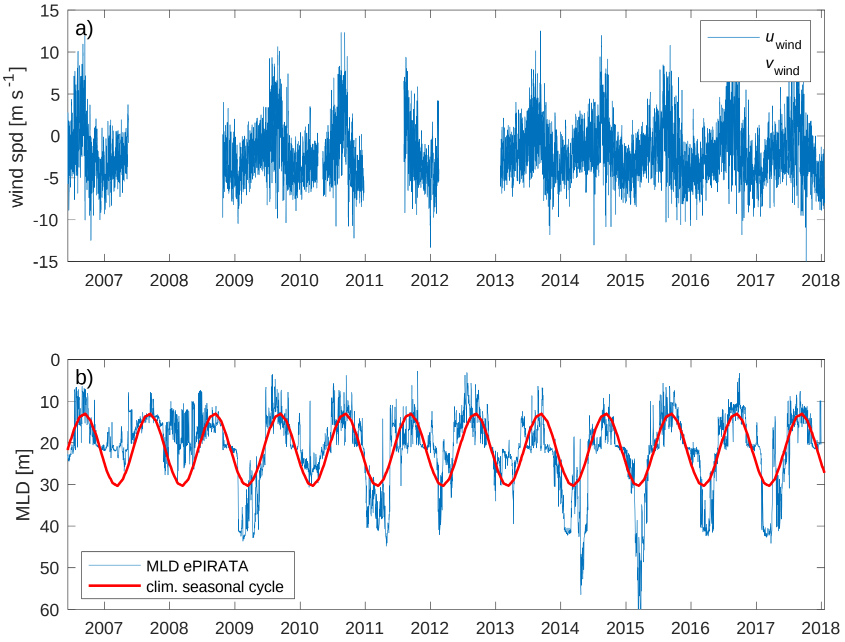

Data input for the slab model

a 4m winds from PIRATA and **b** Mixed layer depth with climatological seasonal cycle constructed from the ePIRATA data set (2006-2017) at 11.5°N, 23°W.

Supplementary Note

WPI at near inertial frequency

Another quantity that is worth considering, in an attempt to quantify the seasonal distribution of energy fed into the ocean at near-inertial frequency, is the so-called wind power input (WPI). WPI is defined as $WPI = \boldsymbol{\tau} \cdot \boldsymbol{u}_{\mathrm{inertial}}$, where $\boldsymbol{\tau}$ is the unfiltered wind stress and $\boldsymbol{u}_{\mathrm{inertial}}$ the near-inertial band-pass filtered ocean velocity. The slab model can represent ML currents fairly well for several inertial cycles after an isolated wind event, but it systematically overestimates WPI due to the lack of damping on very short time scales [1]. Therefore, only direct ML velocity observations should be used (available since November 2015 at 11.5°N, 23°W), to determine the seasonal cycle of WPI. The near-inertial ocean velocity $\boldsymbol{u}_{\mathrm{inertial}}$ is

obtained using a third-order Butterworth bandpass filter retaining frequencies between 0.7*f* and 1.3*f* as in other studies estimating WPI [2, 3].

The longest time scale present in the near-inertial current time series obtained with this band-pass filter is associated with the decay of the inertial oscillations. Therefore, it can be expected that for a sufficiently long time series, the lagged autocovariance of the near inertial current speed should retain only this decay scale expressed as the e-folding timescale of the autocovariance. For the 2-year time series available at the PIRATA buoy at 11.5°N, 23°W, the e-folding and hence the decay scale is 5 days (Fig. S2a), thus it was chosen as the damping time scale for the slab model and linear filter to derive best agreement between model and observations in terms of amplitude and phase of ML velocities for the particular event described in section Modelling the near-inertial ML velocities at the PIRATA buoy site (Fig. 5c).

Wind events trigger a sharp spike in positive WPI during the time the wind vectors and ocean vectors align, which is typically followed by a negative spike, when wind and currents rotate out of phase (Fig. S2b). For comparison, WPI of the slab model is also shown and indicates similar variability as WPI calculated with observed velocities (Fig. S2b). However, in agreement with [1], the cumulative WPI based on slab model velocities overestimates WPI based on observations by a factor of 1.1-1.3 by the end of the 2-year record (Fig. S2c). Nevertheless, even with the overestimated WPI from the slab model, both cumulative estimates of WPI for the years 2016 and 2017 exhibit a similar seasonal evolution: a moderately increasing time-integrated WPI during the first half of the year until June/July, followed by a stronger increase in time-integrated WPI of about 3500-4200 Jm^{-2} during July to September, beyond which the increase drops off again (Fig. S2c). This is in agreement with the analysis presented in the main manuscript showing the seasonal cycle of IA and identifying the second half of the year, and especially July to September, as the period when wind events are most effective in producing a strong inertial ML response (Fig. 6).

Supplementary Figure 2:

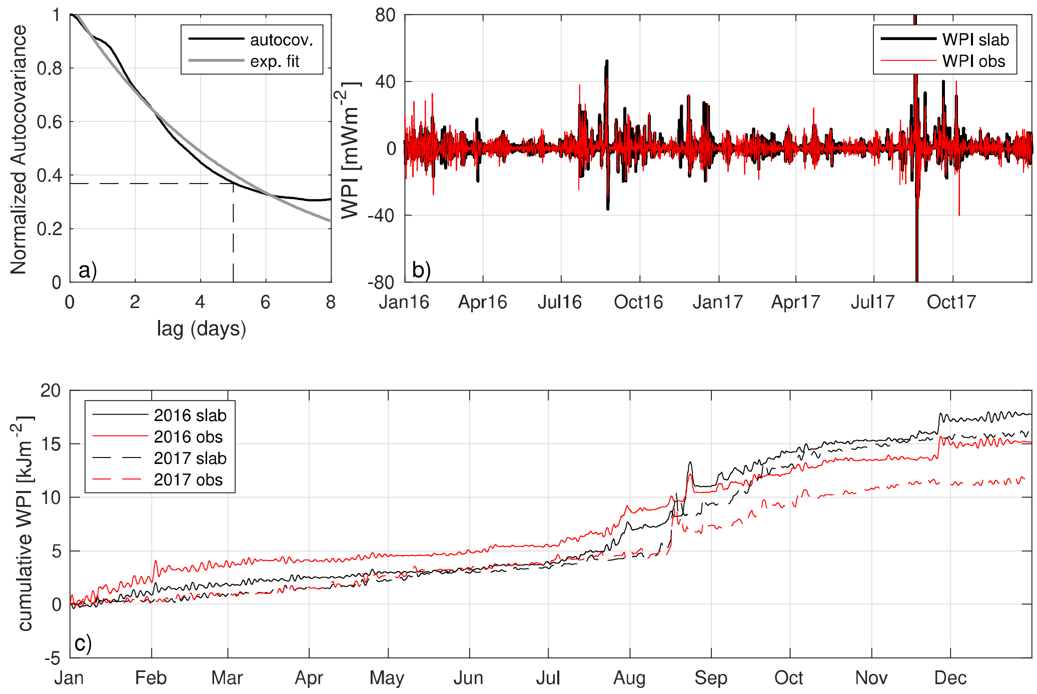

Damping time scale and wind power input

a Normalized lagged autocovariance of near-inertial currents filtered with a 3rd order Butterworth bandpass filter retaining the frequencies between 0.7f and 1.3f based on observed ML velocities at 12m depth from observations at the PIRATA buoy at 11.5°N, 23°W. **b** Wind power input (WPI) time series from observed winds and slab model velocities (black), as well as from observed winds and observed velocities (red), at 11.5°N, 23°W from 2016 to 2017. **c** Time integrated WPI based on slab model (black) and observed velocities (red) for the 2 years of available observational data.

Supplementary Figure 3:

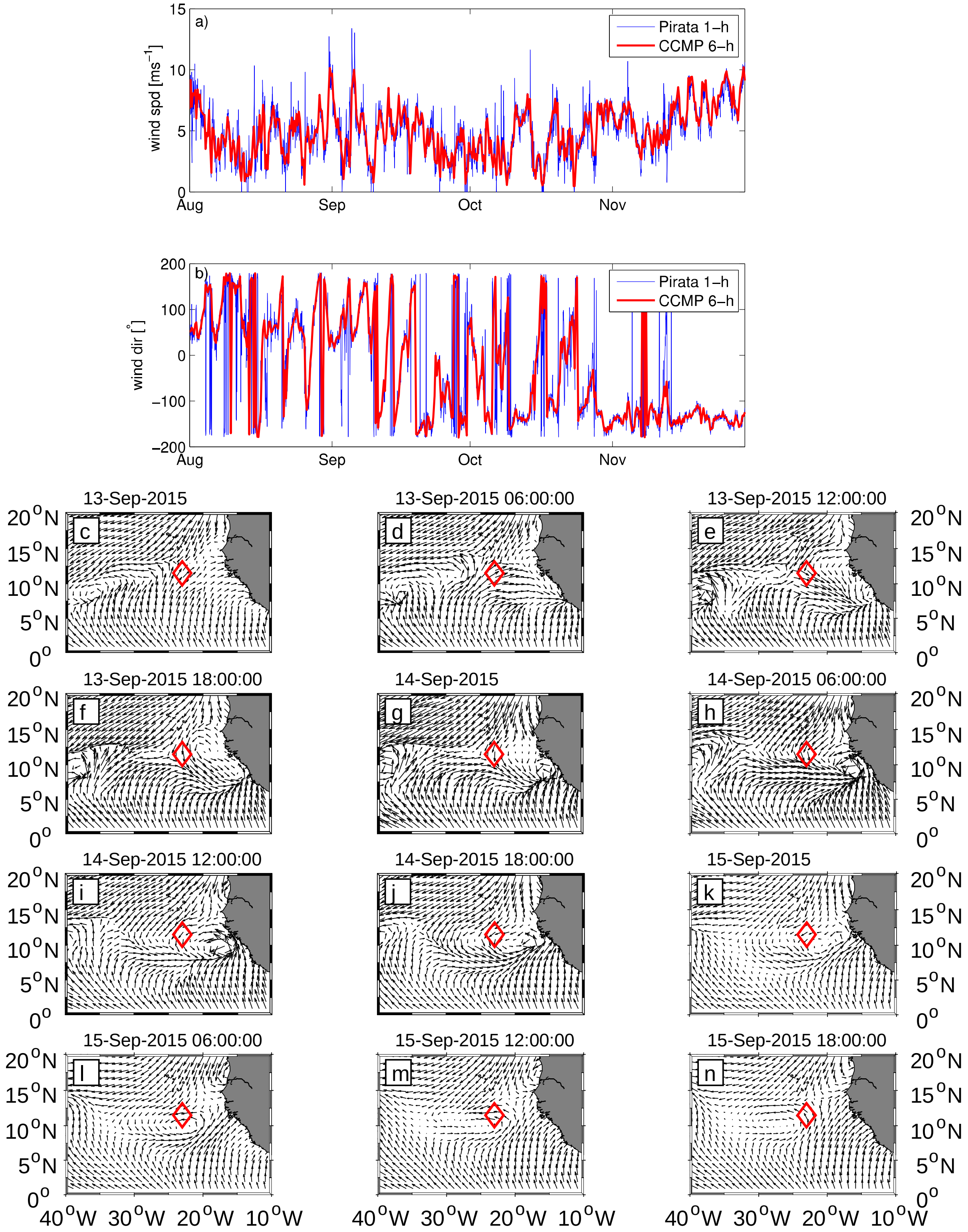

The surface wind field at and around the PIRATA buoy at 11.5°N, 23°W

a-b Comparison of PIRATA (blue) and CCMP (red) wind speed **a** and wind direction **b**. **c-n** Temporal evolution (6 hourly resolution) of the wind field from CCMP in the tropical

Atlantic for the period of 13 - 15 September 2015. The red diamond indicates the position of the PIRATA buoy.

Supplementary Figure 4:

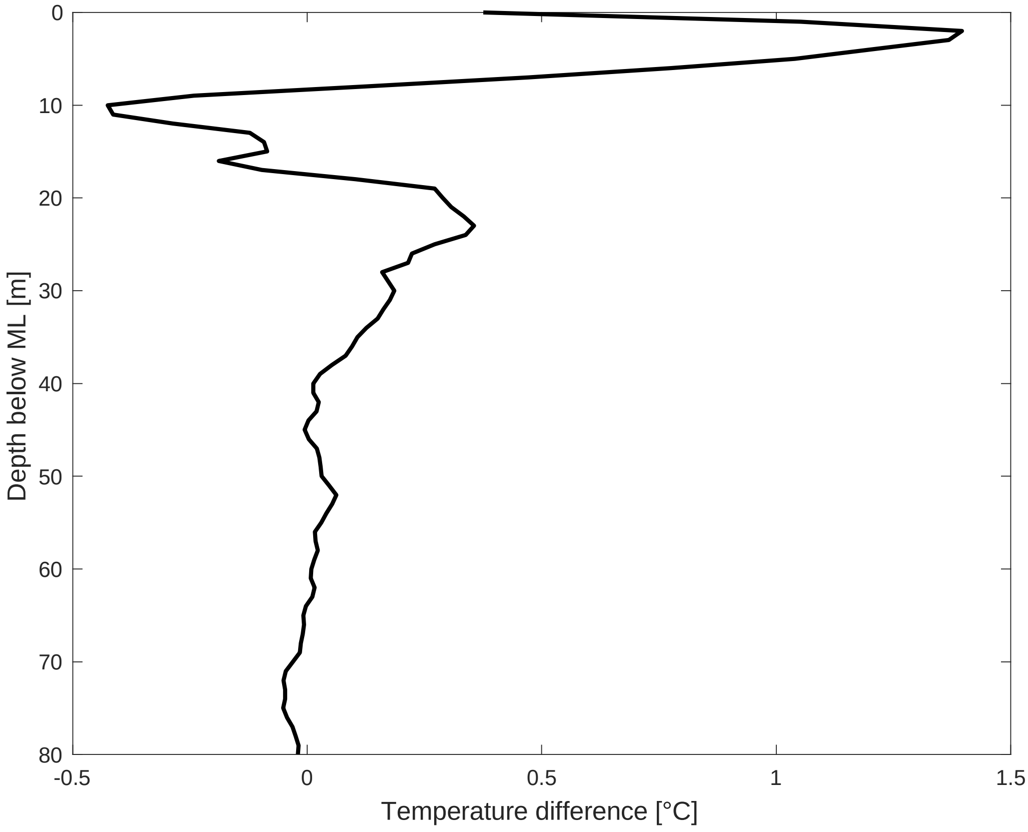

Subsurface temperature evolution during microstructure observations

Temperature difference profile between the last and first 12 microstructure profiles below the mixed layer. The warming and cooling in the upper thermocline above 20m below the ML likely originates from lateral heat advection. Warming between 20m and 40m below the ML is in agreement with the divergence of the vertical diffusive heat flux.

Supplementary References

1. Plueddemann AJ, Farrar JT. Observations and models of the energy flux from the wind to mixed-layer inertial currents. *Deep Sea Research Part II: Topical Studies in Oceanography* **53**, 5-30 (2006).

2. Furuichi N, Hibiya T, Niwa Y. Model-predicted distribution of wind-induced internal wave energy in the world's oceans. *Journal of Geophysical Research: Oceans* **113**, n/a-n/a (2008).

3. Rimac A, von Storch J-S, Eden C, Haak H. The influence of high-resolution wind stress field on the power input to near-inertial motions in the ocean. *Geophysical Research Letters* **40**, 4882-4886 (2013).
